# Transmission pathways and risk factors for sporadic salmonellosis and campylobacteriosis: a source attribution meta-analysis of European case-control studies

**DOI:** 10.1017/S095026882510023X

**Published:** 2025-07-01

**Authors:** Lapo Mughini-Gras, Lena Wijnen, Sara M. Pires, Elisa Benincà, Charlotte Onstwedder, Tine Hald, Eelco Franz, Axel Bonacic Marinovic

**Affiliations:** 1 Institute for Risk Assessment Sciences (IRAS), Utrecht University, Utrecht, The Netherlands; 2 Center for Infectious Disease Control, National Institute for Public Health and the Environment (RIVM), Bilthoven, The Netherlands; 3 National Food Institute, Technical University of Denmark (DTU), Lyngby, Denmark

**Keywords:** campylobacter, case-control study, meta-analysis, salmonella, source attribution

## Abstract

Case-control studies can provide attribution estimates of the likely sources of zoonotic pathogens. We applied a meta-analytical model within a Bayesian estimation framework to pool population attributable fractions (PAFs) from European case-control studies of sporadic campylobacteriosis and salmonellosis. The input data were obtained from two existing systematic reviews, supplemented with additional literature searches, covering the period 2000–2021. In total, 12 studies on *Campylobacter* providing data for 180 PAFs referring to 5983 cases and 13213 controls, and five studies on *Salmonella* providing data for 75 PAFs referring to 2908 cases and 5913 controls, were included. All these studies were conducted in Western or Northern European countries. Both pathogens were estimated as being predominantly linked to food- and waterborne transmission, which explained nearly half of the cases, with *Campylobacter* being mainly attributable to poultry (meat), and *Salmonella* to poultry (eggs and meat) and pig (meat), as specific foodborne exposures. When also considering contact with animals, around 60% of cases could be explained by the larger group of zoonotic transmission pathways. While environmental transmission was also sizeable (around 10%), about a quarter of cases could be explained by factors such as travel, underlying diseases/medicine use, person-to-person transmission and occupational exposure.

## Introduction

In Europe, foodborne infections are responsible for an estimated 23 million cases, 5000 deaths, and 400000 disability-adjusted life years (DALYs) annually [1, 2]. Campylobacteriosis and salmonellosis rank consistently as the first and second most reported zoonoses in Europe [1, 3], accounting for the highest disease burden of foodborne infections on the continent (82000 and 107000 DALYs, respectively) [1, 2]. The estimated annual costs of campylobacteriosis and salmonellosis are also substantial (€ 2.4 and 3 billion, respectively), mostly due to productivity loss and healthcare system use [4, 5].

In principle, foodborne infections are preventable, with shared responsibilities for several actors in the food production chain, from farmers to consumers. Most *Campylobacter* infections are sporadic, and although *Salmonella* is a frequent cause of foodborne outbreaks, most salmonellosis cases do occur sporadically. Both *Campylobacter* and *Salmonella* infections are frequently associated with foodborne transmission, but other transmission routes, such as contact with animals and environmental sources, as well as person-to-person transmission, can also play a role [6–9]. Quantifying the relative contributions of different potential sources of foodborne infections like campylobacteriosis and salmonellosis based on source attribution analyses helps prioritize public health interventions [10, 11]. An example of a highly effective intervention based on a source attribution analysis can be found in New Zealand where the incidence of reported campylobacteriosis was reduced by 54% in 2008 (vs. 2002–2006) after the implementation of interventions targeted at poultry, with a concurrent 74% decline in the fraction of campylobacteriosis cases attributed specifically to poultry as the source of infection [12].

There are several approaches to source attribution of foodborne diseases, which have been critically reviewed elsewhere [10, 11]. Each approach has its own strengths and weaknesses; depending on the type, quality, and quantity of available data, the specific research questions are addressed and the characteristics of the pathogens are attributed. Of the several types of studies aiming at quantifying the sources of sporadic *Salmonella* and *Campylobacter* infections, case-control studies are the most common ones in analytical epidemiological research. In these studies, cases (infected individuals) and controls (asymptomatic and assumed uninfected individuals) are interviewed so that the frequencies of their exposure to several putative risk factors for infection are compared, typically using logistic regression models and with associations expressed as odds ratios (ORs). Case-control studies are particularly valuable to identify risk factors for infection, including potential (food, animal, environmental, and anthroponotic) sources of zoonotic pathogens like *Salmonella* and *Campylobacter*, as well as predisposing factors (e.g., comorbidities, medicine use, etc.) and other exposures (e.g., occupation, travel, etc.) [6–9]. Moreover, when disease occurrence is positively associated with exposure, a population attributable fraction (PAF) for that exposure can be estimated. The PAF is widely used in epidemiology to quantify the potential impact of an exposure on the outcome of interest in a population and is defined as the proportion of cases occurring in the population that is attributable to a specific exposure; that is, the fraction of cases that could theoretically be averted if a perfect intervention would eliminate the exposure in question [13].

In 2012, two systematic reviews of case-control studies of sporadic campylobacteriosis [6] and salmonellosis [7] provided a comprehensive summary of the most important risk factors for these infections. These meta-analyses included 72 studies (38 for campylobacteriosis and 34 for salmonellosis) originating from Europe, North America, and Oceania available in the literature in 2010 (year of the search). For campylobacteriosis, the most important risk factors were international travel, eating undercooked chicken, environment-related factors (e.g., bathing in recreational water), and direct contact with (pet and farm) animals [6]. For salmonellosis, the main risk factors were also travelling abroad, underlying (chronic) diseases, and medicine use, as well as eating raw eggs and eating in restaurants [7]. Other systematic reviews and meta-analyses using slightly different inclusion criteria, time periods, and countries obtained similar results [8, 9]. While such meta-analyses help inform public health efforts, they need to be updated regularly and, ideally, provide pooled epidemiological measures other than only ORs, such as the PAFs, for (groups of) exposures that can be used more directly to attribute the burden of the diseases in question.

Recently, a statistical modelling approach to source attribution meta-analysis within a Bayesian framework has been developed to generate attribution estimates at different levels across the transmission chain for sporadic infections with zoonotic pathogens, including *Salmonella* and *Campylobacter* [14]. This method offers the opportunity to pool the attribution estimates from different (and usually less comprehensive) studies, including PAFs for different exposures from case-control studies, providing an alternative to synthetize quantitative evidence from multiple (yet inevitably incomplete) small-scale studies in a statistically principled way [14]. The aim of the present study was to generate attribution estimates at the level of transmission pathway (e.g. food consumption, contact with animals, etc.) and risk factor within each pathway (e.g. eating chicken meat, contact with dogs, etc.) by applying the aforementioned meta-analytical attribution model on the PAFs extracted from case-control studies of sporadic campylobacteriosis and salmonellosis conducted in Europe and published between 2000 and 2021.

## Methods

### Data collection

The analysis was based on the PAFs extracted from the available European case-control studies, included in two previous systematic reviews focussing on sporadic campylobacteriosis [6] and salmonellosis [7]. These studies were supplemented with additional, more recent studies that had not been included in the earlier reviews, covering a study period from 2000 to 2021. The literature search for these additional studies was built upon the methodology of the earlier systematic reviews, utilizing the same search engines, keywords, relevance, and quality assessment criteria. As such, we refer to the previous systematic reviews [6, 7] for a detailed description of the methodology. In brief, the literature search was conducted in June 2021 using databases such as PubMed, PMC, Science Direct, Ovid, EMBASE, Medline, ISI Web of Science and Web of Science, CAB Direct, CAB international and NAZ. The search employed all possible combinations of (1) general terms related to case-control studies and risk factors and (2) terms specific to *Campylobacter* (or campylobacteriosis) and *Salmonella* (or salmonellosis). Relevance screening of the studies was based on the following inclusion criteria: (1) focus on human disease; (2) focus on *Campylobacter* or *Salmonella*; (3) focus on sporadic (i.e., non-outbreak-related) disease; and (4) use of a case-control study design. The quality of studies was further evaluated using the following criteria: (1) statistical power above 80%; (2) case definition based on laboratory testing; (3) random selection of controls; (4) comparability between cases and controls (in time, space, and underlying population); (5) control for potential confounding factors (i.e., multivariable approach or matching); (6) calculation of ORs and 95% confidence intervals (CI) based on logistic regression. Studies conducted during or after the COVID-19 epidemic (2020 and later) were excluded because the epidemiology of both salmonellosis and campylobacteriosis was significantly impacted by non-pharmaceutical interventions implemented to curb the spread of COVID-19, e.g. [15–18].

Data from the articles that met the eligibility criteria were manually extracted using the same standardized format as in the previous reviews [6, 7]. The extraction information included the year(s) and country of data collection, the age of the study population (adults ≥18 years, children <18 years, or mixed), the number of cases and controls, and the study outcomes: multivariable (adjusted for confounders) ORs and 95% CIs for each statistically significant (*p* < 0.05) risk factor. Additionally, where available, details on the *Campylobacter* species and *Salmonella* serotypes investigated were also recorded.

### Source categorization

The extracted risk factors were organized using a previously established hierarchical framework that classified sources and exposure locations into mutually exclusive source categories [6, 7]. This classification considered the primary animal reservoirs of the pathogens (e.g., broiler chickens, pigs, cattle, etc.), but was primarily structured around potential transmission pathways (e.g., foodborne, waterborne, environmental, etc.) and associated risk factors (e.g., consuming chicken meat or pork, owning a pet, working on a farm, etc.). This approach aligns with insights provided by case-control studies, which typically shed light on infection sources at the exposure level [10, 11, 14, 19, 20]. The risk factors were ultimately grouped into ten main transmission pathways.

1. *Food consumption*: encompassing risk factors related to the consumption of specific food items (e.g., consumption of beef, pork, eggs, etc.), as well as non-water beverages (e.g., milk, juices, etc.).
2. *Food preparation*: including risk factors related to food handling, as well as how or where the consumed food was prepared (e.g., eating at a restaurant, barbecuing, preparing chicken meat at home, etc.).
3. *Hygiene (lack thereof)*: covering risk factors related to general hygiene practices, both within and outside the kitchen (e.g., handwashing habits, cleaning frequency, and the (mis)use of kitchen tools and appliances, etc.)
4. *Water consumption*: including risk factors related to drinking water or its origin (e.g., drinking tap or bottled water, drinking water from a private well or from a natural water body, etc.).
5. *Contact with animals*: including risk factors related to non-occupational contact with live animals or their bodily fluids (excluding consumables like milk), as well as exposure to excreta, fur, hair, feathers, scales, or skin (e.g., contact with dogs, cats, cattle, sheep, reptiles, wildlife, etc.).
6. *Environment*: including risk factors involving exposure to natural or man-made outdoor environments through contact with environmental water, air, mud, soil, or fomites not covered by other transmission pathways. This can also include exposure to environments where animals reside, feed, and defecate (e.g., swimming in open water, fishing, gardening, etc.).
7. *Occupational exposure*: including risk factors related to an individual’s profession or employment sectors (e.g., healthcare work, occupational contact with animals, working in a slaughterhouse, etc.).
8. *Person-to-person transmission*: including risk factors that suggest potential anthroponotic transmission through direct contact with other individuals, their bodily fluids or excreta (e.g., contact with other people with gastrointestinal symptoms, contact with other sick people in general, household size, etc.).
9. *Predisposition (to infection)*: including risk factors related to the use of medicines or the presence of pre-existing (primarily chronic) conditions that could increase susceptibility to infection (e.g., use of gastric antacids, antibiotic use, having Crohn’s disease, etc.).
10. *Travel*: including risk factors related to acquiring the infection while travelling abroad (i.e., outside the country of the study), regardless of the specific transmission pathway involved.

### Modelling

For each pathogen, we aggregated the PAFs of statistically significant risk factors in the case-control studies, grouping them according to the ten predefined transmission pathways (Section “Source categorization”). PAFs were calculated using Miettinen’s formula [21], based on multivariable ORs (used as proxies for relative risks) and the prevalence of exposure among cases as follows:(1)

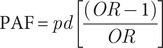

where *pd* is the prevalence of exposure to the risk factor among the cases.

For each pathogen, we applied Bayesian statistical modelling to estimate the proportion of human cases attributable to each transmission pathway. This approach accounted for heterogeneity across studies due to differences in population age, country, and data collection periods.

In each study *s*, we define *ps_i,s_* as the attribution estimate for transmission pathway *i* (with *i = 1,…,Q*, with *Q* being the total number of transmission pathways). Since the set of transmission pathways varied between studies (i.e. not all studies assessed the same pathways), some estimates for certain pathways were missing. These missing values could not be assumed to be zero, meaning that *ps_i,s_* represent partial estimates and may not sum to 1 across all pathways. To address this, we normalized the attribution estimates to obtain a complete set of proportions, denoted as *p_i,s_*, using the following formula:(2)

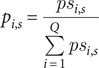

to ensure that 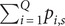 is constrained to 1. This approach is analogous to utilizing a Dirichlet distribution and a multinomial distribution likelihood. However, we opted for the alternative method outlined above due to the presence of missing data in the dataset and the inability of the Bayesian analysis software Just Another Gibbs Sampler (JAGS) to handle partially observed multinomial distribution.

To ensure that *ps_i,s_* values were naturally bounded between 0 and 1, we introduced the parameter *pp_i,s_*, which relates to *ps_i,s_* by the logistic function:(3)

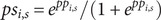



The source attribution parameters *pp_i,s_* have prior normal distributions 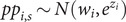 , where *w* and *z* have vague prior normal distributions 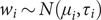 and 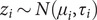 , with mean 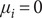 and precision 
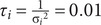
 ( 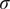 is the standard deviation).

The studies providing data for a specific transmission pathway varied significantly in terms of population age, country, and the years of data collection, all of which could potentially influence the attribution estimate *p_i,s_.* To account for this variability, additional terms were introduced: *b_j,s_*, representing the contribution (in log odds) to the attribution estimate from the different age categories *j (j = 1,…,A*, where *A* being the total number of age categories) and *b_k,s_* representing the contribution (in log odds) from the various countries *k* (*k = 1,…,C*, where C is the total number of countries).

The log odds *b_j,s_* and *b_k,s_* were assumed to follow normal distributions: 
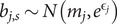
 and 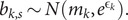 The parameters 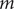 and 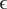 were given non-informative prior normal distributions such that 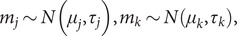 
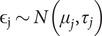
 and 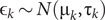 , with mean 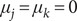 and precision 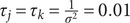 ( 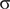 is the standard deviation).

Next, we defined *L* as the total number of unique combinations of *i*, *j, k*, and *s* and introduced the attribution estimate as:(4)

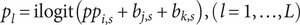

where 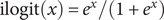 and *p_l_* = *p_i,s_* without heterogeneities between studies. For each unique combination of study, pathway, age group, and country, the number of human cases attributed to each transmission pathway was modelled as being drawn from a binomial distribution:(5)

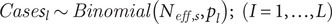

where *N_eff,s_* represents the effective number of human cases in study *s.* As noted earlier, the set of transmission pathways analyzed varied between studies. Consequently, if a study did not account for all potential pathways within a specific age category and country, some observations would be missing. These missing observations had to be imputed for each unobserved pathway in each study. Importantly, these ‘extra’ observations would contribute to increasing the precision of the estimates. To account for this, an effective number of human cases *N_eff,s_* for each study was used in place of the total observed number of cases (*N_total,s_*). Specifically, an additional number of cases (*N_extra,s_*) was added to the observed total, such that: *N_total,s_* as *N_eff.s_* = *N_total,s_* + *N_extra,s_.* The *N*_extra,s_ was sampled stochastically for each study *s* from a Poisson distribution: *N_extra,s_* ~ Poisson(λ*_extra,s_*), where λ*_extra,s_* was drawn from a vague prior distribution: 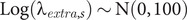 . This framework allowed the model to impute missing pathways using proportions informed by studies that did include those particular transmission pathways.

Since recent studies are more likely to reflect current conditions and provide more relevant information, we assigned greater weight to these studies by reducing the precision ( 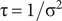 ) of older studies as follows:(6)

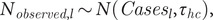

where 
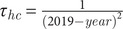
 ; thus, if a study was conducted in 2019: 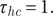 Consequently, *Cases_l_* were modelled to follow the actual estimated number of cases observed in a study (*N_observed,l_*), with declining precision as the publication year dated further back from 2019.

The actual number of observed cases attributed to each transmission pathway cannot be measured directly, so an estimate, *N_observed,l_*, was used instead. For each transmission pathway, the total number of cases in a study/age category/country combination is denoted as *N_total,l_.* However, not all cases can be classified because the set of studied pathways often does not account for all possible existing transmission pathways. As a result, the number of human cases per pathway in a given study/age category/country combination that can provide information is represented as *N_observed,l_*, which is smaller than *N_total,l_.* This inherently reduces the accuracy of the estimate *p_i,l_,* where *i* denotes the transmission pathway (i = 1*,…,Q_l_*), and *Q_l_* represents the number of studied transmission pathways in study *s.* Let(7)

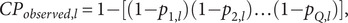

where *CP_observed_* represents the cumulative probability of a human case in study/age category/country *l* being attributed to one of the transmission pathways that were observed in that study*. N*ote that *Q_l_ ≤ Q*, where Q is the total number of possible transmission pathways. Using this, the total number of cases in the study *s* that can be classified into one of the observed transmission pathways is expressed as:(8)

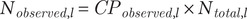



The calculations were carried out using Markov Chain Monte Carlo (MCMC) sampling. The model was implemented and executed in JAGS (v4.3) [22], interfaced with the statistical programming language R (v4.03). Five parallel chains were run for 100,000 iterations, including a burn-in phase of 10,000 iterations to allow for stabilization. Convergence of the model was assessed visually by inspecting the mixing of the posterior distributions across the chains.

Ethical approval was not necessary, as this is a meta-analytical modelling study that synthesizes data from previously published studies. The data used in this analysis consisted solely of anonymized, aggregate statistics obtained from the primary studies.

## Results

### Descriptive results

When adding the studies selected from the previous reviews [6, 7] to those identified in the additional search, the total number of studies included in the meta-analysis was 12 for *Campylobacter* and five for *Salmonella*. Details of these studies are reported in Table 1. In total, the 12 studies on *Campylobacter* provided data for 180 PAFs referring to 5983 cases and 13213 controls, while the five studies on *Salmonella* provided data for 75 PAFs referring to 2908 cases and 5913 controls. All these studies were conducted in Western or Northern European countries. As shown in Table 2, the 180 PAFs for *Campylobacter* could be divided into ten transmission pathways and over 49 risk factors within these pathways. The largest number of PAFs for *Campylobacter* (*n* = 63, 35%) fell within the food consumption pathway, followed by contact with animals (*n* = 34, 19%). The 75 PAFs for *Salmonella* could be divided over nine transmission pathways and over 23 risk factors within these pathways (Table 3). Also for *Salmonella*, the largest number of PAFs (*n* = 33, 44%) fell within the food consumption pathway, followed by contact with animals (*n* = 10, 13%).

**Table 1.** Overview of the case-control studies of sporadic human campylobacteriosis and salmonellosis included in the source attribution meta-analysis

References	Country	Number of Cases	Number of Controls	Number of PAFs
*Campylobacter*				
Bless et al. [23]	Switzerland	159	280	8
Carrique-Mas et al. [24]	Sweden	126	270	6
Danis et al. [25]	Ireland	197	296	12
Doorduyn et al. [26]	Netherlands	1019	3119	26
Gallay et al. [27]	France	285	286	8
Kapperud et al. [28]	Norway	212	422	20
Kuhn et al. [29]	Denmark	556	2117	54
MacDonald et al. [30]	Norway	995	1501	13
Mossong et al. [31]	Luxembourg	415	624	13
Rosner et al. [32]	Germany	1812	3983	11
Schonberg-Norio et al. [33]	Finland	100	137	4
Wingstrand et al. [34]	Denmark	107	178	5
Total		5983	13213	180
*Salmonella*				
Doorduyn et al. [35]	Netherlands	533	3119	8
MacDonald et al. [36]	Norway	389	1500	12
Rettenbacher-Riefler et al. [37]	Germany	416	711	33
Ziehm et al. [38]	Germany	1017	176	4
Ziehm et al. [39]	Germany	553	407	18
Total		2908	5913	75

**Table 2.** Pooled attribution estimates for sporadic human campylobacteriosis to different transmission pathways and contribution of specific risk factors within each pathway based on the meta-analysis of population attributable fractions of case-control studies conducted in Europe between 2000 and 2021

Transmission pathway/exposure	Number of PAFs^a^	Pooled mean attribution estimate	95% Confidence Interval	
Food consumption	63	29.6%	16.6%	43.6%
Beef	8	9.8%	8.9%	10.7%
Chicken meat	21	14.0%	13.0%	15.0%
Unpasteurized dairy products	3	1.2%	0.8%	1.7%
Fish & shellfish	1	2.6%	1.8%	3.5%
Fruit & vegetables	10	7.6%	6.8%	8.4%
Game meat	1	2.4%	0.8%	5.3%
Overall meat	5	16.1%	14.7%	17.6%
Other unspecified food products	1	16.0%	14.4%	17.7%
Pork	2	3.5%	2.0%	5.4%
Poultry meat	3	10.1%	9.2%	11.1%
Tripe	1	1.5%	0.3%	3.9%
Turkey meat	1	10.1%	7.3%	12.9%
Undercooked or raw meat	6	5.0%	4.3%	5.8%
Food preparation	22	8.6%	3.7%	14.5%
Eating out	7	31.2%	29.2%	33.1%
Grill/barbecue	7	19.3%	17.5%	21.2%
Handled poultry meat at home	4	19.0%	17.4%	20.6%
Handled raw meat at home	1	13.3%	11.7%	15.2%
Picnic	1	3.3%	2.0%	4.9%
Street food	2	14.0%	10.7%	17.4%
Hygiene (lack of)	3	8.2%	1.1%	23.1%
Kitchen hygiene	2	51.8%	47.8%	63.3%
Wash hands	1	48.2%	36.7%	52.2%
Water consumption	11	7.7%	2.4%	17.9%
Bottled water	1	21.9%	17.6%	27.2%
Own water supply or well	4	11.2%	8.9%	14.2%
River, stream, or lake water	3	8.0%	6.2%	10.2%
Tap water outside home	1	27.4%	13.6%	33.3%
Undisinfected water	2	31.4%	26.2%	39.7%
Contact with animals	34	9.6%	4.1%	17.9%
Cats	4	12.5%	10.1%	14.5%
Cattle	4	8.1%	6.3%	10.1%
Dogs	8	13.7%	12.5%	15.1%
Unspecified farm animals	4	13.9%	13.0%	15.4%
Other unspecified animals	7	13.7%	12.4%	15.2%
Poultry	4	11.6%	7.4%	14.3%
Sheep	2	12.7%	8.4%	14.6%
Wild animals	1	13.7%	11.9%	15.2%
Environment	14	9.5%	2.9%	19.2%
Fishing	1	6.1%	3.1%	9.7%
Unspecified outdoor activities	1	14.5%	10.0%	19.5%
Contact with sand or soil	5	44.5%	40.6%	48.8%
Swimming	7	34.9%	30.5%	40.4%
Occupation	4	3.9%	0.2%	17.4%
With animals or raw meat	3	51.4%	44.8%	64.1%
In childcare	1	48.6%	35.9%	55.2%
Person-to-person	4	5.2%	0.9%	13.6%
Contact person with diarrhoea	3	55.7%	0.1%	99.7%
Contact with other sick people	1	44.3%	0.3%	99.9%
Predisposition	12	7.6%	1.8%	19.2%
Antibiotic use	1	25.8%	13.4%	40.7%
Chronic diseases	6	24.3%	17.2%	31.9%
Proton pump inhibitor use	5	50.0%	40.1%	59.3%
Travel	13	10.2%	3.6%	22.5%
Abroad (overall)	4	35.5%	31.3%	40.1%
Inside Europe	1	14.6%	7.7%	21.8%
Outside Europe	6	40.9%	35.6%	46.5%
Unspecified destination	2	9.0%	5.7%	12.9%

a Number of population attributable fractions (PAFs) extracted from the studies and included as data points in the analysis.

**Table 3.** Pooled attribution estimates for sporadic human salmonellosis to different transmission pathways and contribution of specific risk factors within each pathway based on the meta-analysis of population attributable fractions of case-control studies conducted in Europe between 2000 and 2021

Transmission pathway/exposure	Number of PAFs^a^	Mean attribution estimate	95% Confidence Interval	
Food consumption	33	6.2%	4.2%	8.4%
Beef	2	5.2%	3.6%	7.2%
Eggs	6	18.0%	15.6%	20.7%
Fruit and vegetables	4	10.1%	8.2%	12.3%
Lamb and mutton meat	2	3.6%	2.3%	5.2%
Other unspecified food products	1	5.5%	3.8%	7.5%
Pork	11	23.6%	20.8%	27.8%
Poultry meat	3	14.1%	11.8%	16.8%
Undercooked or raw meat	4	19.9%	12.2%	24.2%
Food preparation	7	12.4%	2.7%	30.2%
Eating out	6	81.9%	49.4%	98.2%
Grill/barbecue	1	18.1%	1.8%	50.6%
Hygiene	4	9.9%	1.6%	30.5%
Kitchen hygiene	2	32.7%	27.4%	38.1%
Wash hands	2	67.3%	61.9%	72.6%
Water consumption	2	13.9%	2.4%	38.4%
Contact with animals	10	13.7%	5.3%	26.2%
Cats	3	21.8%	17.9%	26.1%
Cattle	2	8.1%	5.1%	11.9%
Dogs	3	17.0%	13.6%	20.9%
Other unspecified animals	2	53.1%	47.5%	58.8%
Environment	5	9.9%	1.6%	29.0%
Sand or soil	3	45.4%	2.3%	95.9%
Swimming	2	54.6%	4.1%	97.7%
Occupation	2	11.6%	1.3%	34.9%
Predisposition	9	12.4%	4.5%	25.5%
Antibiotic use	3	43.3%	35.3%	50.1%
Proton pump inhibitor use	6	56.7%	49.9%	64.7%
Travel	3	10.1%	1.9%	26.5%

a Number of population attributable fractions (PAFs) extracted from the studies and included as data points in the analysis.

### Overall attributions at transmission pathway level

**Figure 1.**
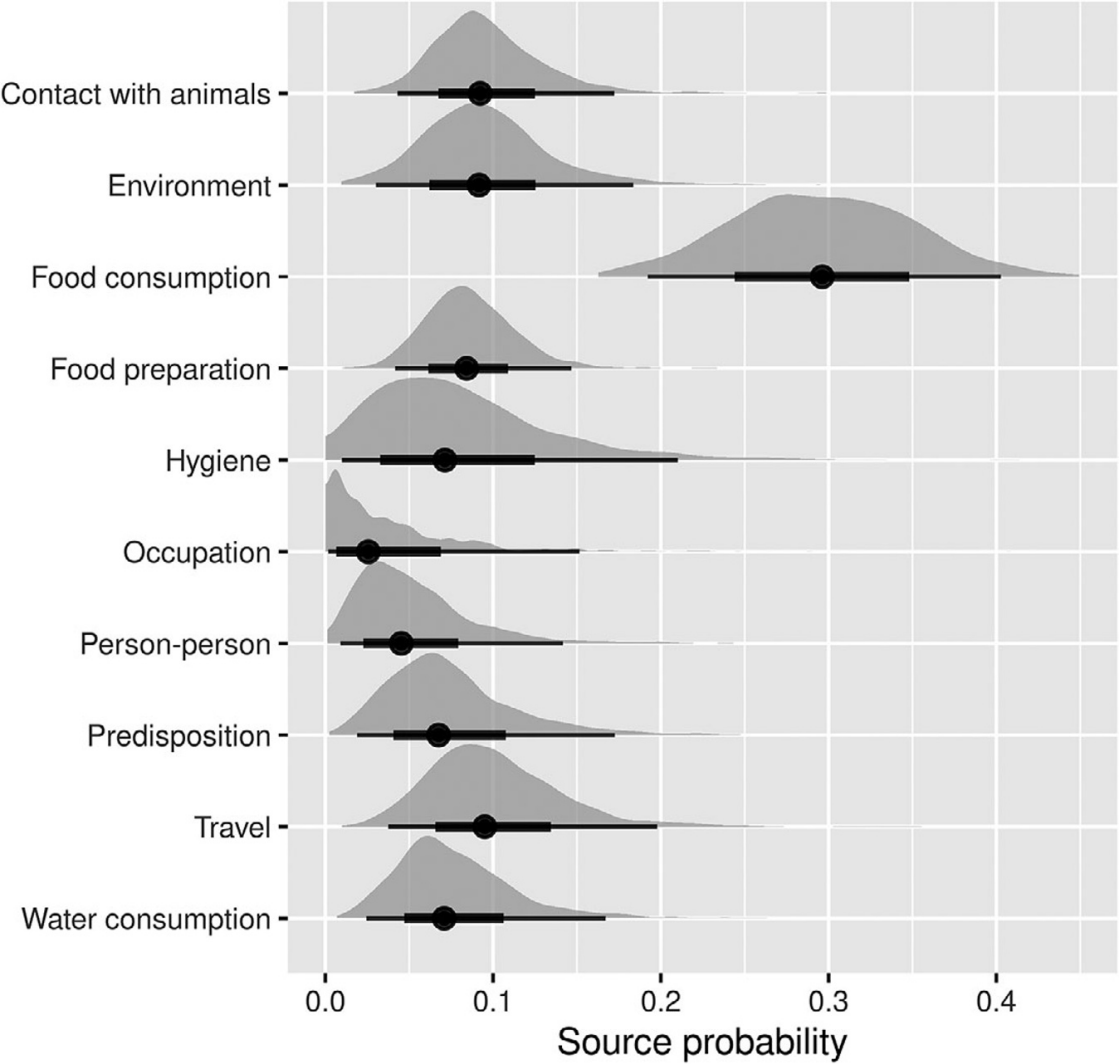
Pooled attribution estimates (source probability distributions) for sporadic human campylobacteriosis to different transmission pathways based on the meta-analysis of population attributable fractions of case-control studies conducted in Europe between 2000 and 2021. The width of the half-violin plots represents the density of data at each source probability value. The averages (dots) of the source probabilities are mutually exclusive (summing to 1) and reflect the proportion of human cases attributable to each transmission pathway. The thick and thin bars indicate the interquartile range and 1.5x interquartile range, respectively.

As shown in Figure 1, the main transmission pathway for human campylobacteriosis was estimated to be food consumption, accounting for 29.6% (95%CI 16.6–43.6%) of cases. Among the other food- and waterborne-related transmission pathways, this was followed by food preparation (8.6%, 95%CI 3.7–14.5%), hygiene deficiencies (8.2%, 95%CI 1.1–23.1%), and water consumption (7.7%, 95%CI 2.4–17.9%). Of the other (non-foodborne/non-waterborne-related) transmission pathways, contact with animals accounted for 9.6% (95%CI 4.1–17.9%) of cases, followed by the environment (9.5%, 95%CI 2.9–19.2%), while travel, predisposing factors, person-to-person transmission, and occupational exposure accounted for 10.2% (95%CI 3.6–22.5%), 7.6% (95%CI 1.8–19.2%), 5.2% (95%CI 0.9–13.6%), and 3.9% (95%CI 0.2–17.4%) of cases, respectively.

**Figure 2.**
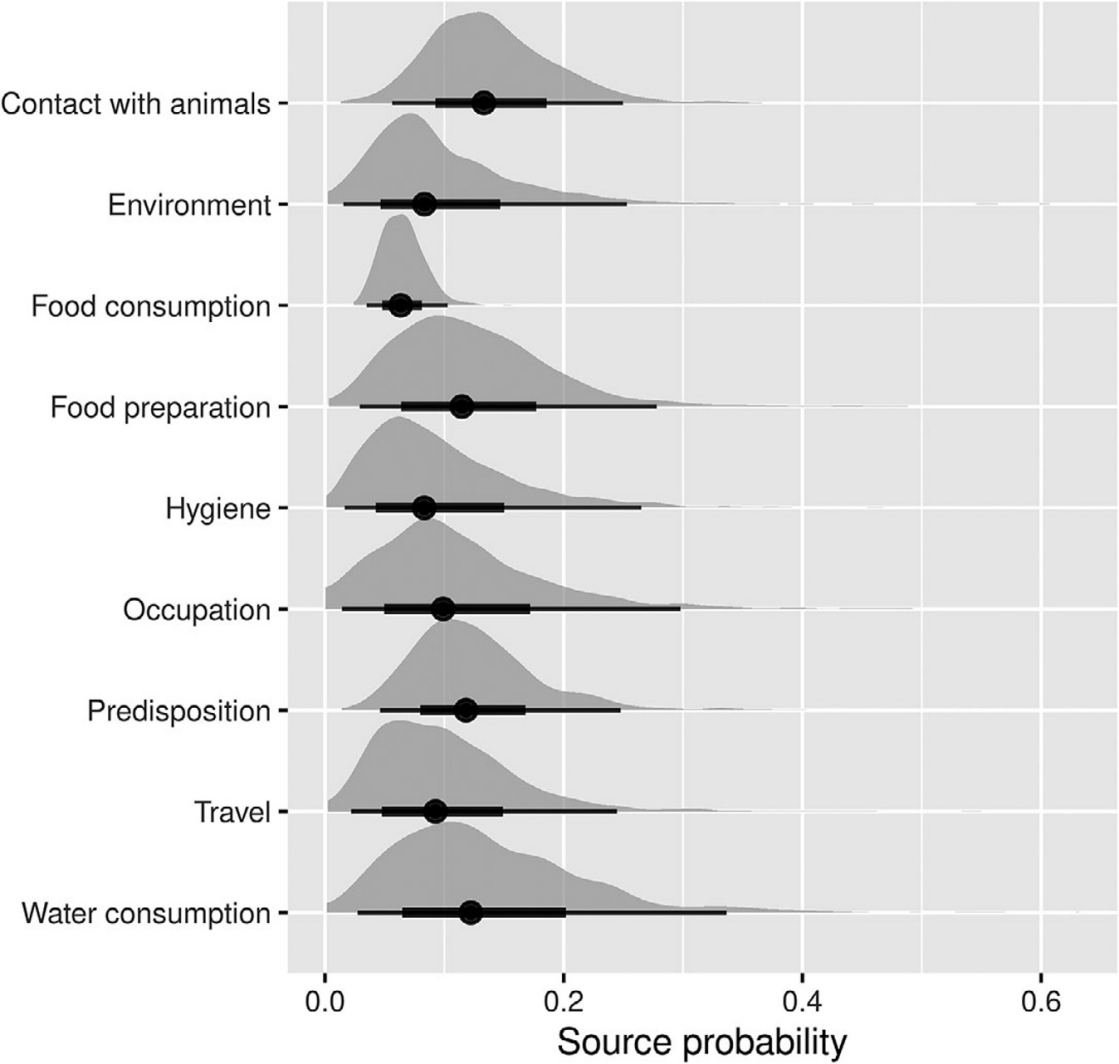
Pooled attribution estimates (source probability distributions) for sporadic human salmonellosis to different transmission pathways based on the meta-analysis of population attributable fractions of case-control studies conducted in Europe between 2000 and 2021. The width of the half-violin plots represents the density of data at each source probability value. The averages (dots) of the source probabilities are mutually exclusive (summing to 1) and reflect the proportion of human cases attributable to each transmission pathway. The thick and thin bars indicate the interquartile range and 1.5x interquartile range, respectively.

Among the food- and waterborne-related transmission pathways of human salmonellosis (Figure 2), water consumption, food preparation, hygiene deficiencies, and food consumption accounted for 13.9% (95%CI 2.4–38.4%), 12.4% (95%CI 2.7–30.2%), 9.9% (95%CI 1.6–30.5%), and 6.2% (95%CI 4.2%–8.4%) of cases, respectively. Of the other (non-foodborne/non-waterborne related) transmission pathways, contact with animals accounted for 13.7% (95%CI 5.3–26.2%), occupational exposure for 11.6% (95%CI 1.3–34.9%), and the environment for 9.9% (95%CI 2.4–31.3%), while travel and predisposing factors accounted for 10.1% (95%CI 1.9–26.5%) and 12.4% (95%CI 4.5–25.5%) of cases, respectively.

### Attributions at transmission pathway level by pathogen subtype and age group

For *Campylobacter*, subtyping categorization was based on species. Attribution estimates were synthesized using data from studies that reported risk factors specifically for *Campylobacter jejuni* or *Campylobacter coli* separately, for both species combined (*C. jejuni/coli*), or for *Campylobacter* spp. when species-level differentiation was not possible (Supplementary Material, Table S1). Not all pathways were represented in the attributions for each species, given the low number of studies (only two) providing estimates for *C. jejuni* or *C. coli* separately. Yet, these species-specific attributions, as well as those for *C. jejuni/coli* and for all *Campylobacter* spp., were similar to the overall ones (Figure 1). For food consumption, estimates tended to be lower when species were combined or unspecified. Age groups could be categorized as children, adults, and all (both children and adults, undifferentiated). Attribution estimates were generally similar across age categories, with some differences being observed for food consumption and person to person between children and adults.

For *Salmonella*, subtyping categorization was based on the serotypes Enteritidis or Typhimurium (separately), other serotypes than Enteritidis and Typhimurium, or as *Salmonella enterica* in general when serotype-level differentiation was not possible (Supplementary Material, Table S2). Also, here, not all pathways could be represented for each serotype, given the low number of studies providing estimates for Enteritidis or Typhimurium separately. Yet, it could be observed that serotype Typhimurium had a relatively higher contribution from the environment and food consumption than Enteritidis, whereas Enteritidis had a relatively higher contribution from predisposing factors. Age groups could be categorized as children, adults, or both (undifferentiated). Attribution estimates were generally similar across age categories, with a few differences observed for hygiene, food consumption and preparation, and travel between children and adults.

### Overall attributions at the risk factor level

The pooled attribution estimates for campylobacteriosis and salmonellosis broken down by risk factor within each pathway are reported in Tables 2 and 3, respectively. For *Campylobacter*, within the food consumption pathway, the risk factors with the highest attributions were meat of unspecified origin (16%), chicken meat (14%), turkey meat (10%), unspecified poultry meat (10%), and beef (10%). Eating out was the main risk factor in the food preparation pathway and drinking undisinfected/unpurified water in the water consumption one. The importance of the risk factors in the pathway contact with animals was comparable (all between 8% and 14%), while within the environmental pathways, touching sand or soil, and open water swimming, were the most important ones (45% and 35%, respectively). Using PPI (50%) and travelling outside Europe (41%) were the most important risk factors for the predisposition and travel pathways, respectively. The other pathways contained only two risk factors each, and cases were evenly attributed to them (Table 2).

For salmonellosis (Table 3), because some pathways only included one risk factor, it was not possible to break them down any further. Therefore, the attribution estimates at the risk factor level were generated only for the food consumption, food preparation, hygiene, contact with animals, environment, and predisposition pathways. Within the food consumption pathway, the risk factor with the highest attribution was pork (24%), followed by undercooked/raw meat of unspecified origin (20%) and eggs (18%). Among the risk factors included in the contact with animal pathway, contact with unspecified animal species was the most important (53%), followed by cats (22%) and dogs (17%). The other pathways contained only two risk factors each and were evenly attributed between them, except for the food preparation pathway, where the risk factor eating out dominated (82%), and for the hygiene pathway where factors related to handwashing were the most important (67%).

## Discussion

This study quantifies the relative contributions of multiple transmission pathways and exposures to sporadic human infections with *Campylobacter* and *Salmonella* in Europe in 2000–2021. Most PAFs were related to food consumption, followed by animal contact, for both *Campylobacter* and *Salmonella*. Food- and water-related routes together accounted for about 54% of campylobacteriosis and 42% of salmonellosis cases. These estimates are relatively lower than those in other studies, likely due to the numerous pathways considered here, since the share of each source decreases as more sources are included. When adding contact with animals to the food- and water-borne routes, approximately 64% of campylobacteriosis and 56% of salmonellosis cases were attributed to ‘zoonotic transmission routes.’ The environment also plays a role by mediating the spread of *Salmonella* and *Campylobacter* between animals and humans. While the environment can be a reservoir for certain *Salmonella* strains [35, 40, 41], this is less likely for *Campylobacter*, which usually performs poorly outside hosts. Indeed, the fate of *Campylobacter* in the environment is more a matter of survival rather than growth [42–44], although some strains show greater environmental resilience [45]. Including environmental transmission also raises attribution to 74% for campylobacteriosis and 66% for salmonellosis. European source attribution studies based on microbial subtyping that estimated the contribution of the environment to *Salmonella* or *Campylobacter* infections are limited, but available data for *Campylobacter* suggest a contribution of around 10% [46], consistent with our findings.

Predisposition and travel, while included in the analysis, are not true transmission routes, but rather umbrella categories entailing various exposures in which virtually all routes (food, water, animals, environment, etc.) might play a role. We found predisposition and travel to account for a substantial share of cases (18% for campylobacteriosis and 22% for salmonellosis). Although the specific sources of infection in these groups cannot be identified due to limited detail (e.g., only ‘travel’ reported in the original studies), their contribution via increased susceptibility to infection or increased exposure to (potentially more virulent) pathogen strains is clearly significant. Identifying the share of non-travel-related (i.e., domestically acquired) cases remains important, as these are the ones domestic food safety policies can directly address. The same applies to predisposition, which reflects the inherent contribution of high-risk groups. Travel-related cases and those with predisposition may also be foodborne, but reducing their burden requires different interventions, such as targeted advice for travellers or people with comorbidities, among others. When the contributions of predisposition and travel are added to the aforementioned ones, approximately 91% of campylobacteriosis cases and 89% of salmonellosis cases can be explained.

Case-control studies focus on sporadic cases, capturing exposures that may differ from (and be less specific than) those identified in outbreak investigations. These cases typically stem from routine diagnostics of individuals with more severe, symptomatic gastroenteritis and therefore reflect the more serious end of the clinical spectrum. As a result, the identified attributions and risk factors pertain mainly to severe infections and may not fully represent all cases. However, serological studies suggest that risk factors for exposure to campylobacter align closely with those for clinically overt disease [47]. Still, exposure patterns may vary for individuals more susceptible to severe illness (e.g., immunocompromised persons, pregnant women), who are often advised to avoid high-risk foods. From a public health standpoint, identifying the sources of severe infections is particularly important, as these cases place the greatest strain on healthcare systems.

A relatively small fraction of cases was attributable to person-to-person transmission (around 5%, quantifiable only for campylobacter) and occupational exposure. Person-to-person transmission is considered minor for both (non-typhoidal) salmonella and campylobacter, as these zoonotic infections are generally short lived with minimal asymptomatic carriage (0.05% for *Salmonella* and 0.19% for *Campylobacter* in a 2016 Dutch study [48]), meaning humans are not a significant reservoir. This aligns with our findings and previous estimates, such as a 3% contribution of person-to-person spread for *Campylobacter* among returning travellers [49]. Together, travel, predisposition, person-to-person transmission, and occupational exposure account for about a quarter of cases, underscoring the multifactorial nature of transmission and the role of non-foodborne routes [6–9].

Stratifying estimates by age group and pathogen subtype yielded results largely consistent with overall findings. For *Campylobacter*, adults showed relatively larger contributions from food consumption and preparation, while hygiene and person-to-person transmission were more prominent in children, likely reflecting typical household roles and hygiene behaviours. These patterns were not observed for salmonella, casting some doubt on their plausibility, especially given the pathogens’ similar epidemiology and limited data for stratified analysis. Stratification by *Campylobacter* species (*jejuni* and *coli*) showed minimal differences, except possibly in food-related pathways. For *Salmonella*, Typhimurium was more associated with environmental exposure than Enteriditis, in line with current knowledge [8].

Breaking down attribution estimates by exposure provided more detail within each transmission pathway and largely confirmed existing knowledge. For example, for *Campylobacter*, 73% of food-related cases were linked to meat, mainly poultry (34%), unspecified meat (21%), and beef (10%), aligning with known risk sources [6, 9, 31, 32, 34, 46]. For *Salmonella*, major food exposures included poultry (32% in total), especially eggs (18%) and poultry meat (14%), as well as pork (24%), consistent with known sources of serotypes Enteritidis and Typhimurium, respectively [7, 8, 50]. Eating out was a key contributor in the food preparation category, especially for *Campylobacter*, supporting previous findings of higher risk when consuming chicken outside the household [31, 51–53] due to increased chance of exposure to (higher doses of) specific *Campylobacter* strains different from those to which people are usually exposed at home [31, 46, 51–53]. In the animal contact pathway, pets (dogs and cats) accounted for 26% of *Campylobacter* and 39% of *Salmonella* cases, highlighting significant zoonotic risks. However, reverse transmission (from humans to animals) or shared infection sources in the household (e.g., homemade pet food, kitchen scraps) cannot be excluded [54]. Defining the directionality of transmission from associative (epidemiological) studies, such as case-control studies, is therefore challenging. Regarding environmental sources, contact with soil and swimming in open waters were major exposures for both *Campylobacter* and *Salmonella*. As these pathogens are widespread in the outdoor environment, these exposures appear plausible. However, whether the environment serves as a reservoir or merely a transmission route remains uncertain.

The attribution estimates presented here were generated using a previously developed Bayesian meta-analytical source attribution model [14], which synthetizes PAFs extracted from case-control studies. This approach enables integration of data from diverse studies, helping to overcome common meta-analysis challenges such as differences in design, populations, and definitions, which also limited the number of studies included here. Nonetheless, the model effectively leveraged available case-control data to produce attribution estimates across multiple pathways and exposures — a level of detail often unattainable in individual source attribution studies, which typically rely on expert elicitation to fill data gaps [10, 14]. As no expert elicitation was used for anchoring, the analysis was based solely on empirical data. While this strengthened its data-driven foundation, it also limits completeness, as certain sources could not be included due to a lack of data. However, the model remains flexible and can be updated as new studies become available. Synthesizing data while preserving original uncertainties is the best way to maximize the value of information scattered across the literature. We chose to include only statistically significant multivariable associations for several reasons: (1) non-significant ORs add uncertainty to pooled estimates; (2) differences in multivariable models across studies risk including poorly adjusted or unstable results; (3) despite sample size effects, statistical significance helps prioritize associations with stronger evidence; and (4) although missing data is a limitation, it is not outweighed by including weak or uncertain findings.

The model also differs from the previous version [14] by jointly estimating attributions for both pathways and their exposures using the same input data, offering two connected levels of source contribution within the transmission chain. While informed by literature data, the goal was synthesis rather than a systematic review per se, meaning outcomes depend on the scope and quality of the included studies, and different research questions can be answered with different data inputs using this method. Categorization of pathways and exposures was pragmatically chosen based on prior studies, data availability, and research focus, but can be adapted by recalculating the percentages in Tables 2 and 3. This flexibility allows for re-categorization when different groupings are more relevant, for example, by intervention type, such as combining all food-related exposures regardless of preparation or consumption context. This is also useful given the overlap between categories like ‘food preparation’ and ‘hygiene,’ where poor hand hygiene is closely linked to mishandling raw meat or inadequate grilling practices. In contrast, exposures like eating out or consuming street food involve risks less directly tied to consumer’s personal hygiene.

We also excluded studies conducted during the COVID-19 pandemic years. A global review study on *Campylobacter* showed a significant drop in reported incidence of campylobacteriosis in 22 out of 26 European countries [55]. Similarly, studies from the Netherlands showed decreases in both salmonellosis and campylobacteriosis incidence during COVID-19 [15, 56]. Studies in other countries have also shown reductions in campylobacteriosis and salmonellosis incidence associated with non-pharmaceutical measures being implemented against COVID-19 [18, 57, 58]. This decrease was mainly linked to travel restrictions and lockdowns with different degrees of stringency, as well as altered exposure patterns (e.g. fewer social gatherings), healthcare-seeking behaviours, and healthcare capacity, which might therefore lead to distorted pictures of the actual epidemiological situation of the pathogen. For these reasons, we did not include studies conducted during the COVID-19 years.

In conclusion, applying a Bayesian meta-analytical model to combine attribution estimates from various European case-control studies on campylobacteriosis and salmonellosis yielded detailed, biologically plausible estimates of the relative contributions of multiple transmission pathways and exposures. This approach also offers valuable insights into how consensus estimates can be derived from available empirical data from epidemiological studies.

## Supporting information

10.1017/S095026882510023X.sm001 Mughini-Gras et al. supplementary material Mughini-Gras et al. supplementary material

## Data Availability

Data from this study are available in previous publications as described in the manuscript or from the authors upon request.

## Supplementary material

The supplementary material for this article can be found at http://doi.org/10.1017/S095026882510023X.
