# Supplementary material for: Transmission pathways and risk factors for sporadic salmonellosis and campylobacteriosis: a source attribution meta-analysis of European case-control studies
Source: Epidemiol Infect. 2025 Jul 1;153:e77. doi: 10.1017/S095026882510023X (PMC12247009; doi:10.1017/S095026882510023X)
Supplement: Mughini-Gras et al. supplementary material [file S095026882510023Xsup001.docx]

**Supplementary Material**

**Table S1.** Pooled attribution estimates (in percentage, with 95% confidence intervals) for sporadic human campylobacteriosis to different transmission pathways. The estimates are differentiated per *Campylobacter* species and age group and are based on the meta-analysis of population attributable fractions of case-control studies conducted in Europe between 2000 and 2021.

|  | ***Campylobacter* species** | | | | **Age group** | | |
| --- | --- | --- | --- | --- | --- | --- | --- |
|  | ***C. jejuni* (2;22)#** | ***C. coli* (2;13)#** | ***C. jejuni/coli** (4;34)#** | **Unspecified species** (7;111)#** | **Children (3;17)#** | **Adults (2;3)#** | **Unspecified age*** (11;160)#** |
| Contact with animals | 9.9 (1.8-26.6) | 14.1 (2.7-36.1) | 24.3 (8.8-41.2) | 9.9 (3.0-21.7) | 11.0 (6.2-17.3) | 8.4 (1.0-30.1) | 8.5 (4.6-13.8) |
| Environment | 18.9 (4.5-44.0) | 12.0 (2.4-30.8) | 13.1 (2.7-30.6) | 8.7 (2.4-19.3) | 6.0 (2.1-11.7) | 8.6 (0.5-31.4) | 15.4 (7.3-27.0) |
| Food consumption | 35.4 (23.4-39.3) | 38.7 (15.5-47.0) | 15.2 (9.3-22.6) | 25.1 (9.4-45.9) | 21.4 (9.5-37.1) | 31.2 (19.6-41.5) | 28.6 (15.9-42.0) |
| Food preparation | 16.4 (4.2-34.9) | 20.1 (4.2-43.7) | 16.0 (6.5-29.8) | 7.9 (2.9-14.7) | 6.8 (3.9-10.3) | 8.0 (0.9-25.1) | 10.3 (6.0-15.8) |
| Hygiene | 2.9 (0.3-12.2) | n/a | n/a | 12.4 (2.1-32.7) | 10.2 (0.4-45.7) | 8.1 (0.2-33.3) | 8.1 (1.5-24.3) |
| Occupation | 2.7 (0.3-11.7) | n/a | 6.8 (0.4-23.1) | 6.4 (0.3-21.1) | 7.1 (0.0-43.7) | 6.0 (0.0-37.0) | 2.1 (0.4-8.7) |
| Person-person | 3.8 (0.6-14.6) | n/a | n/a | 5.8 (0.7-17.7) | 8.1 (0.3-36.7) | 4.6 (0.9-12.0) | 3.8 (0.8-9.8) |
| Predisposition | 10.1 (2.2-28.4) | 15.1 (3.1-36.3) | n/a | 6.2 (1.3-15.9) | 9.4 (0.5-36.3) | 7.7 (2.5-17.5) | 5.6 (1.7-12.8) |
| Travel | n/a | n/a | 12.5 (1.4-34.0) | 9.0 (1.4-24.6) | 12.0 (2.7-29.3) | 9.0 (0.8-32.8) | 10.0 (3.2-25.4) |
| Water consumption | n/a | n/a | 12.1 (6.6-19.4) | 8.6 (1.1-27.0) | 8.0 (1.2-24.2) | 8.3 (0.8-30.5) | 7.6 (2.2-17.8) |

*Estimates derived from studies that include *Campylobacter jejuni* and *C. coli*, but do not distinguish between them. These estimates are not the result of combining the previous two columns.

**Estimates derived from studies that do not distinguish between *Campylobacter* species at all. These estimates are not the result of combining the previous three columns, but rather represent a synthesis of PAFs from analyses where species differentiation was not possible.

***Estimates derived from studies that do not distinguish between children and adults. These estimates are not the result of combining the previous two columns, but rather represent a synthesis of PAFs from analyses where age differentiation was not possible.

#Number of studies and number of PAFs used for the estimates.

n/a = not available

**Table S2.** Pooled attribution estimates (in percentage, with 95% confidence intervals) for sporadic human salmonellosis to different transmission pathways. The estimates are differentiated per *Salmonella* serotype and age group and are based on the meta-analysis of population attributable fractions of case-control studies conducted in Europe between 2000 and 2021.

|  | ***Salmonella* serotype** | | | | **Age group** | | |
| --- | --- | --- | --- | --- | --- | --- | --- |
|  | **Enteritidis (2;7)#** | **Typhimurium (3;16)#** | **Other serotypes* (1;4)#** | **Unspecified serotype** (4;48)#** | **Children (1;18)#** | **Adults (1;4)#** | **Unspecified age*** (3;53)#** |
| Contact with animals | n/a | n/a | 22.8 (14.1-33.0) | 15.0 (7.4-26.0) | 12.7 (4.0-29.1) | 14.1 (1.3-44.9) | 12.8 (4.2-30.4) |
| Environment | 12.4 (2.4-29.9) | 22.0 (4.8-50.3) | 20.5 (14.1-33.0) | 8.4 (1.5-26.9) | 12.0 (0.6-52.6) | 12.8 (0.5-56.9) | 6.3 (2.1-13.9) |
| Food consumption | 10.5 (2.2-27.2) | 22.6 (7.1-43.7) | 14.2 (7.1-22.8) | 6.7 (4.5-9.4) | 7.0 (3.3-11.9) | 3.5 (1.6-6.1) | 7.1 (4.9-9.9) |
| Food preparation | 29.0 (8.1-55.5) | 24.3 (5.4-50.7) | n/a | 12.7 (2.3-34.0) | 13.3 (0.9-50.1) | 7.6 (1.7-19.6) | 15.5 (3.3-40.2) |
| Hygiene | n/a | n/a | n/a | 12.8 (2.1-37.6) | 3.8 (0.6-14.0) | 12.9 (0.4-56.8) | 17.1 (2.9-47.4) |
| Occupation | n/a | 17.3 (3.1-46.2) | n/a | 11.5 (1.5-36.6) | 13.2 (0.4-54.0) | 13.9 (0.4-58.2) | 8.8 (1.5-27.2) |
| Predisposition | 27.9 (7.1-53.5) | 13.8 (5.5-25.8) | n/a | 10.3 (1.8-28.3) | 13.5 (1.2-49.5) | 11.4 (4.9-22.9) | 10.2 (4.4-20.5) |
| Travel | 20.3 (4.0-43.3) | n/a | n/a | 8.6 (1.2-26.9) | 11.2 (0.7-41.6) | 8.7 (1.8-24.2) | 9.2 (1.6-27.6) |
| Water consumption | n/a | n/a | 42.5 (29.5-56.7) | 13.8 (1.9-42.7) | 13.9 (0.7-49.8) | 13.3 (0.7-55.1) | 12.9 (3.3-33.1) |

*Estimates derived from studies that include *Salmonella* serotypes other than Enteritidis and Typhimurium. These estimates are not the result of combining the previous two columns.

**Estimates derived from studies that do not distinguish between *Salmonella* serotypes at all. These estimates are not the result of combining the previous three columns, but rather represent a synthesis of PAFs from analyses where serotype differentiation was not possible.

***Estimates derived from studies that do not distinguish between children and adults. These estimates are not the result of combining the previous two columns, but rather represent a synthesis of PAFs from analyses where age differentiation was not possible.

#Number of studies and number of PAFs used for the estimates.

n/a = not available
